# Supplementary material for: Germinated Buckwheat: Effects of Dehulling on Phenolics Profile and Antioxidant Activity of Buckwheat Seeds
Source: Foods. 2021 Apr 1;10(4):740. doi: 10.3390/foods10040740 (PMC8066582; doi:10.3390/foods10040740)
Supplement: Supplementary file 1 [file foods-10-00740-s001.pdf]

Supplementary Table S1. Characteristics of the phenolic compounds in buckwheat extracts as analysed by LC-MS.

	Rt (min)	ESI - Precursor ion	ESI - Fragment ion	Standard LOQ ng/column	Standard LOD ng/column	Sample LOQ ng/column	Semple LOD ng/column	Recovery (%)
Orientin	10,66	447,20	327,20	0,25	0,08	0,52	0,18	93,12
Isoorientin	10,49	447,20	327,20	0,79	0,24	1,23	0,51	96,20
Rutin	11,34	609,20	301,20	0,08	0,03	0,18	0,07	95,31
Vitexin	11,27	431,20	311,20	0,12	0,04	0,15	0,07	93,47
Catechin	3,96	289,10	139,10	0,45	0,13	0,83	0,31	94,33
Epicatechin	7,78	289,10	139,10	0,70	0,21	0,92	0,31	95,98
Hyperin	11,37	463,10	301,10	1,01	0,30	1,11	0,34	94,70
p-coumaric acid	9,30	163,10	119,10	0,53	0,16	0,78	0,27	93,77

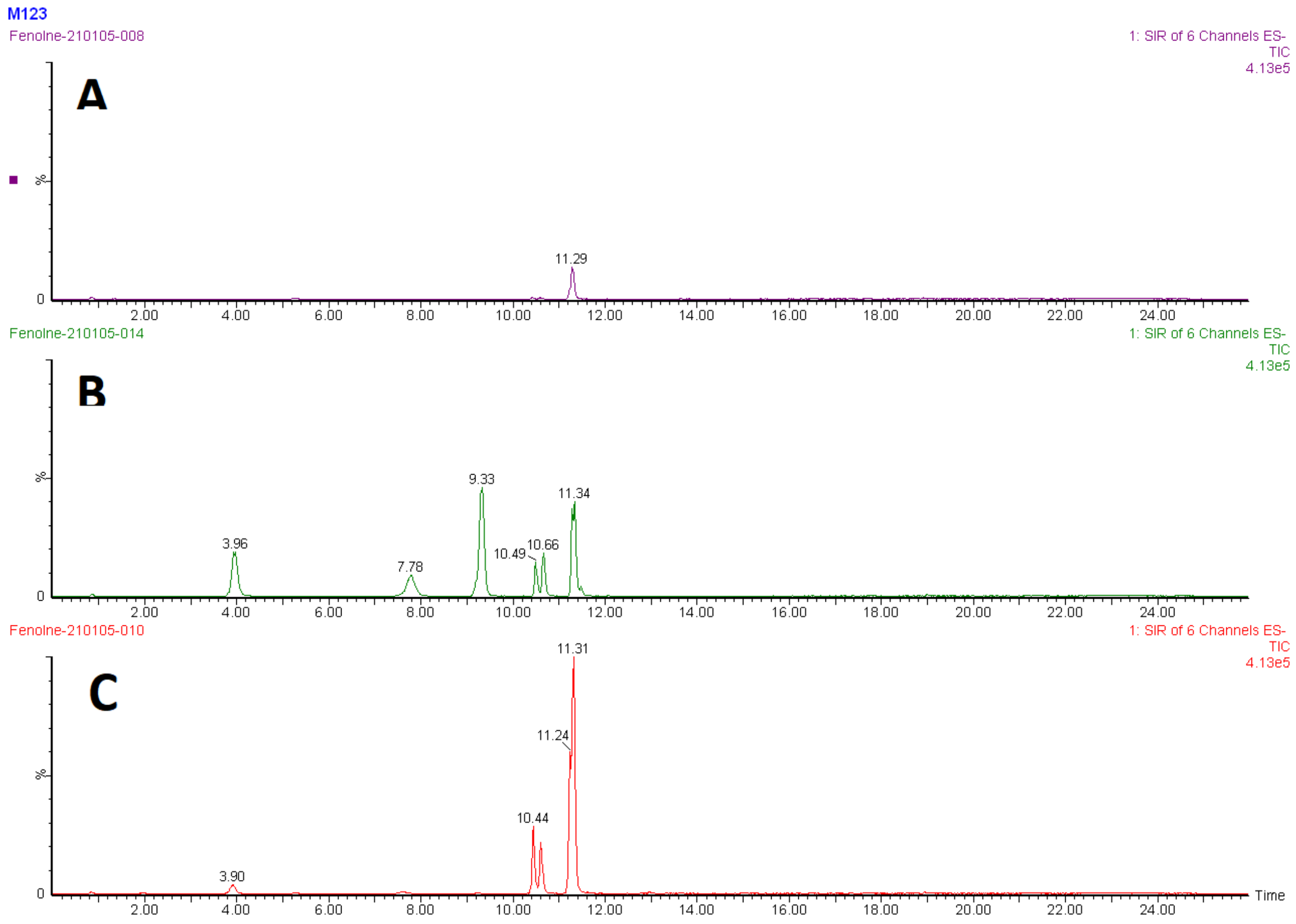

Supplementary Figure S1. Representative mass chromatogram of (A) nongerminated hulled buckwheat extract, (B) nongerminated hulled buckwheat extract spiked with standards and (C) 96 h germinated hulled buckwheat extract.
